# A Comparative Evaluation of the Bonding Strength, Marginal Adaptation, and Microleakage of Dental Cements in Prosthodontics: An In Vitro Comparative Study

**DOI:** 10.7759/cureus.65534

**Published:** 2024-07-27

**Authors:** Fahad Hussain Alhamoudi, Rajesh Vyas, Sunil K Vaddamannu, Lujain Ibrahim N Aldosari, Abdulkhaliq Ali F Alshadidi, Simran Kaur Aulakh, Bhumika Kamal Badiyani, Amit Kumar

**Affiliations:** 1 Department of Dental Technology, College of Applied Medical Science, King Khalid University, Abha, SAU; 2 Department of Prosthodontics, College of Dentistry, King Khalid University, Abha, SAU; 3 Department of Dentistry, Y.M.T. Dental College and Hospital, Kharghar, IND; 4 Department of Public Health Dentistry, Interdental Multispeciality Dental Clinic, Mumbai, IND

**Keywords:** prosthodontics, microleakage, marginal adaptation, bonding strength, resin cement, zinc phosphate cement, resin-modified glass ionomer cement, dental cement

## Abstract

Background: The primary function of dental cement is to seal and support prosthodontic restorative materials. Proper selection of the dental cement contributes to the clinical success of the restoration.

Methods: A total of 166 molar tooth samples were prepared to simulate the type of tooth commonly found in prosthodontic practice. Each sample was restored using one of the tested dental cement materials employing a prestabilized methodology. The performance of resin-modified glass ionomer cement (RMGIC) (GC Fuji PLUS Capsule, GC America, Alsip, IL), zinc phosphate cement (ZPC) (Dentsply Sirona, Charlotte, NC), and resin cement (RC) (RelyX ARC, 3M ESPE, Saint Paul, MN) in bonding strength, marginal adaptation, and microleakage was evaluated and compared. The bonding strength, marginal adaptation, and microgroove were tested using specific established methodologies. The outcomes were then analyzed using statistical analyses for means and standard deviations to compare different types of dental cement.

Results: The total outcome shows that the highest bonding strength with the highest mean was the resin cement, rating 24.8 MPa, followed by RMGIC and ZPC at 20.5 and 18.9 MPa, respectively. The marginal adaptation scores indicate that RC had the highest score at a mean of 4, followed by ZPC at 3.2 and RMGIC at 2.5. The dye penetration measurements in millimeters revealed that ZPC had a penetration of 0.31 mm, RMGIC had a penetration of 0.25 mm, and RC had the least penetration at 0.20 mm. The results of the statistical data analysis show significant differences between the dental cements in bonding strength and marginal adaptation.

Conclusion: In conclusion, resin cement demonstrated superior performance in bonding strength, marginal adaptation, and resistance to microleakage compared to RMGIC and zinc phosphate cement. These findings highlight the importance of selecting resin cement for achieving optimal clinical outcomes in prosthodontic restorations.

## Introduction

Restorations in prosthodontics reinstate function and aesthetics, giving the patient total confidence in regaining the use of the dentition [1]. The selection of dental cement is one of the most critical components for the success of prosthodontic restorations since it acts as an adhesive interface between the restoration and the tooth structure. The composition of dental cement varies widely: it ranges from the old traditional cement like zinc phosphate cement (ZPC) to the newly introduced ones like resin-based formulations, such as resin-modified glass-ionomer cement (RMGIC) and resin cement (RC) [2,3]. Cement has its properties and uses. Hence, some of the considerations are necessary to take it seriously for better results in clinical practice.

Bond strength is another property of longevity and strength in dental restorative materials. These are most typically measured as the parameters defining the performance of cement in service: masticatory force resistance and debonding resistance [4]. Resin-based cement is characterized by high retention force sources, namely chemical adhesion and micromechanical interlocking with dental hard tissues [5]. Most conventional cements depend on the form of a mechanical retention, like ZPC, and hence possess a lowered retention force. Understanding bonding strength is indispensable to making the best material and technique choice for many clinical applications.

Marginal adaptation is the amount of restoration that fits in with its surrounding tooth structure. Bad marginal adaptation is the leading cause of microleakage, secondary caries, and other periodontal problems [6]. Generally, resin-based cement has shown better marginal adaptation than conventional cement; therefore, the margin discrepancies will be lesser in resin-based cement. On the contrary, with conventional cement like ZPC, there will be more margin discrepancies; therefore, precise clinical procedures are required in such cases to gain optimized adaptation. Margin analysis of dental cements gives a clear indication of their performance during clinical procedures and long-term stability [7]. Microleakage is defined as the passage of fluids and bacteria both inside and outside a restorative margin through the potential space between the tooth and restorative material in a manner that adversely influences the restoration and the residual tooth structure below it. Resin-based cements have a more significant sealing potential for the areas of the restoration margin; the degree of microleakage will be reduced, and that, in turn, will lead to the long-term success of the restorations [8]. The classic cements that offer more microleakage are those with lower adhesion; at the same time, moisture and contamination allow the settlement and spread of cariogenic bacteria. Microleakage assessment plays an important role in estimating the clinical performance of dental cement and is a way to avoid the risk of postoperative complications [9].

Much of the literature has been done on dental cement, but more patterns of work based on comparative studies to evaluate the performance of dental cement among the critical parameters of bonding strength, marginal adaptation, and microleakage have to be carried out [10-12]. Such studies provide crucial information for clinicians and researchers to develop proper refinement in selecting the cement and optimizing the clinical protocol. This study will further contribute to the existing scientific knowledge base by offering a systematic guide on the effectiveness of all dental cement used in prosthodontic cementation and, therefore, guide evidence-based practice.

## Materials and methods

To maintain the reliability and uniformity of the materials used in this study, a very precise procedure for preparing samples was performed. To imitate prepared tooth stumps commonly seen in prosthodontic practice, 166 dental molar teeth were made. The sample size was calculated using n = 2σ^2^(Z_α/2​ _+ Z_β​_)^2^​ / Δ^2^, where n is the sample size per group, σ is the estimated standard deviation of the measurements, Z_α/2​ _is the critical value of the standard normal distribution at the desired significance level (α), for a two-tailed test, Z_β_​ is the critical value of the standard normal distribution for the desired power (1 - β), and Δ is the minimum difference between means that you want to detect (effect size).

Each specimen was made from standardized artificial teeth composed of a resin composite material consistent with natural tooth structure. The first step involved careful shaping and contouring of the resin composite material to resemble the anatomy of a prepared tooth stump. Precise trimming and carving were done to achieve the required measurements and morphology representative of clinical situations while ensuring that common features such as occlusal contours, axial walls, and marginal ridges were duplicated to have relevance in clinical practice. The axial reduction was standardized to 1.5 mm to provide adequate space for the restorative material while preserving the tooth's structural integrity and ensuring convergence of 6° for retention and resistance. Occlusal reduction was standardized to 2.0 mm to provide sufficient clearance for the restorative material, particularly for materials requiring a thicker layer for strength, such as all-ceramic restorations, while mimicking clinical practice for both functional and esthetic purposes. High-precision rotary instruments and diamond burs, combined with digital calipers and depth gauges, were employed to ensure reductions were accurate within ±0.1 mm of the intended measurements. All operators involved in the preparation process underwent extensive training, including theoretical instruction, practical demonstrations, and supervised practice sessions until consistent results were achieved. Each prepared tooth was subjected to rigorous quality control measures, including inspection under a stereomicroscope to verify the accuracy of the finish line and reduction; specimens that did not meet standards were reprepared or discarded.

**Figure 1:**
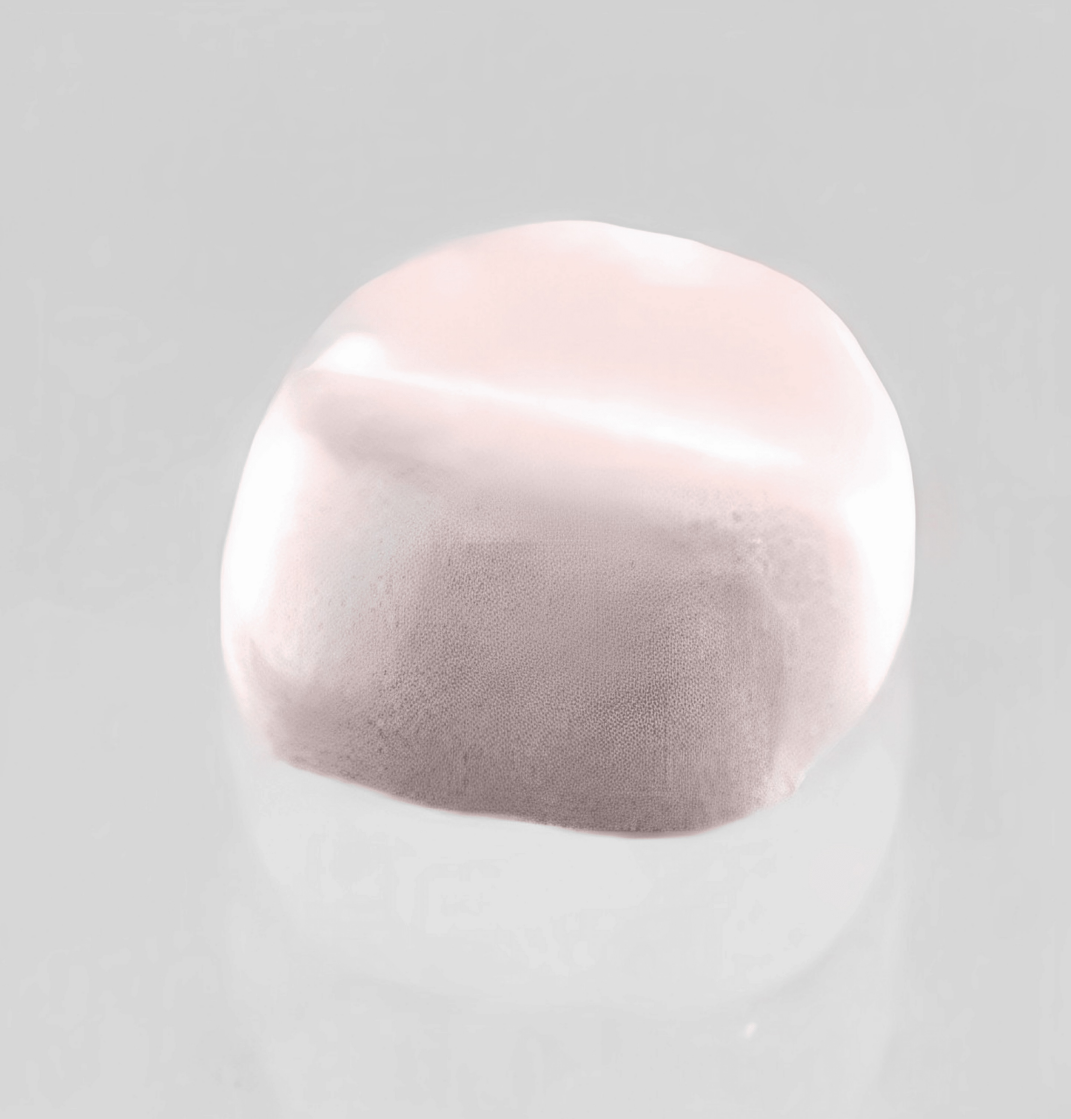
Tooth prepared for the cementation of the crown

**Figure 2:**
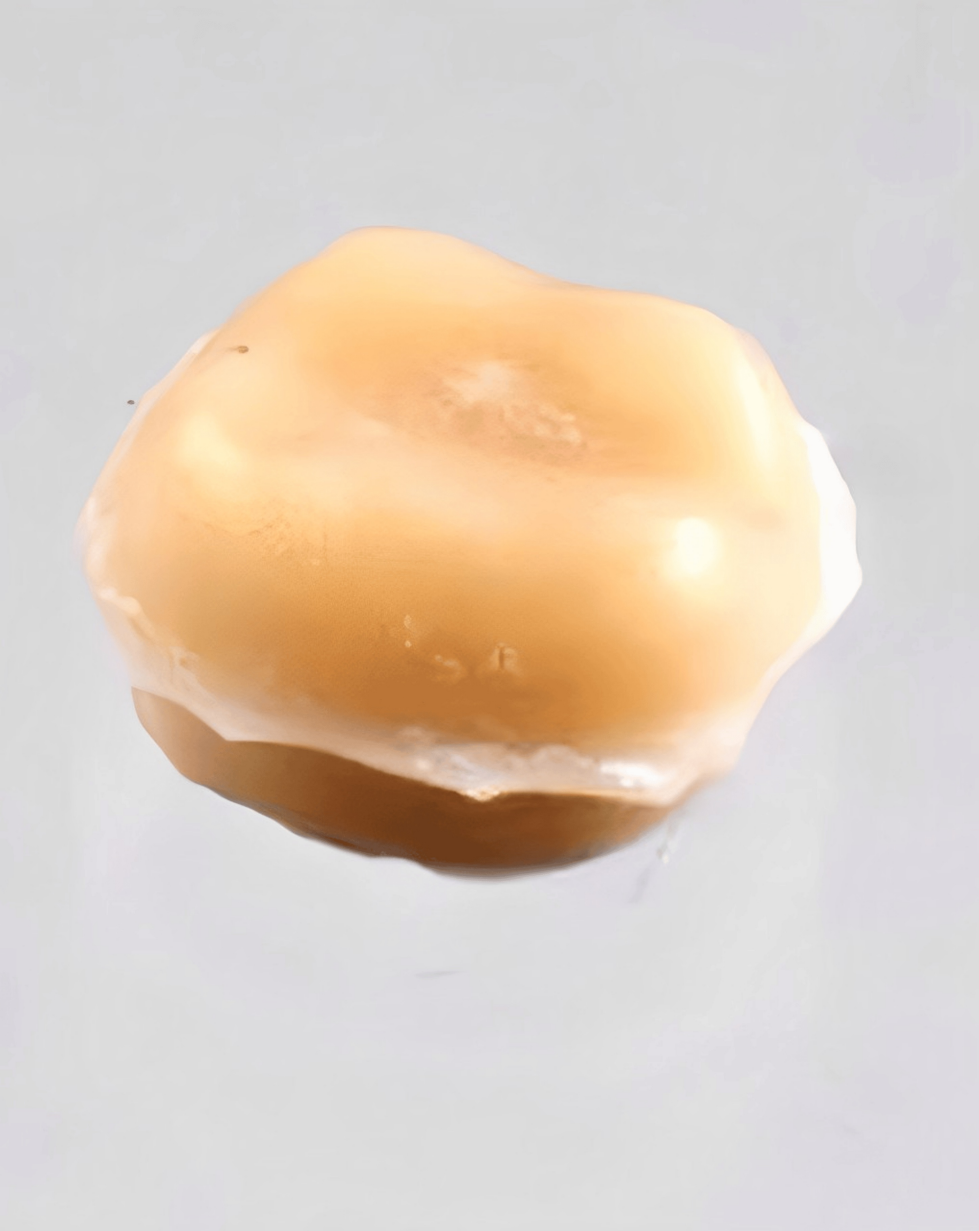
Crown cemented with dental cement to check the marginal adaptation

After shaping, all teeth that had been prepared underwent thorough cleaning and polishing by removing any surface debris or contaminants that could compromise their integrity or interfere with cement bonding. Every specimen was magnified and inspected under a microscope to ensure no visible defects or imperfections were present. Once cleaned and polished, these samples were stored in an environment where their properties would not degrade before cementation; this involves controlling temperature and humidity levels, among other factors. Throughout the sample preparation process, there was strict adherence to standard operating procedures (SOPs), ensuring that similar things were done according to set rules every time. The study followed specific rules for applying dental cement on each tooth sample to ensure exactness and consistency among all samples. Each of the teeth was allotted one of three dental cements: RMGIC (GC Fuji PLUS Capsule, GC America, Alsip, IL), ZPC (Dentsply Sirona, Charlotte, NC), and RC (RelyX ARC, 3M ESPE, Saint Paul, MN). To achieve the best consistency and homogeneity, the cements were mixed properly before use as recommended by the manufacturer. This included accurately measuring powder and liquid parts of the cement and mixing them well with calibrated equipment. While maintaining the integrity of the mixtures, care was taken to ensure the correct ratio between powder and liquid components was achieved without introducing air (Figures 1, 2).

Those who applied mixed dental cement onto a prepared tooth stump were skilled operators who had knowledge of prosthodontics procedures. Spatulas or syringe applicators, which are special tools, were used to place and manipulate them precisely where required within such small spaces. The whole area that had been prepared on top of each tooth stump received an even coat thickness of around 50-100 µm, which was maintained through careful application by these individuals so that there would be uniformity across all specimens.

Throughout the cement application process, steps were followed according to strict protocols set up by organizations such as ISO 29042-3:2008(E). This was done to minimize variation between operators and enhance reproducibility in different labs worldwide. In order not to compromise any bonding strength due to excess amounts or voids left behind during the placement phase, which may have occurred because some people did not follow instructions correctly, meticulous working practice should take place while dealing with adhesive systems anyway. For instance, if there is a discrepancy noticed during inspection that could affect adhesion properties, then it must be corrected immediately; otherwise, other samples will also need rework.

Bonding strength measurement

**Figure 3:**
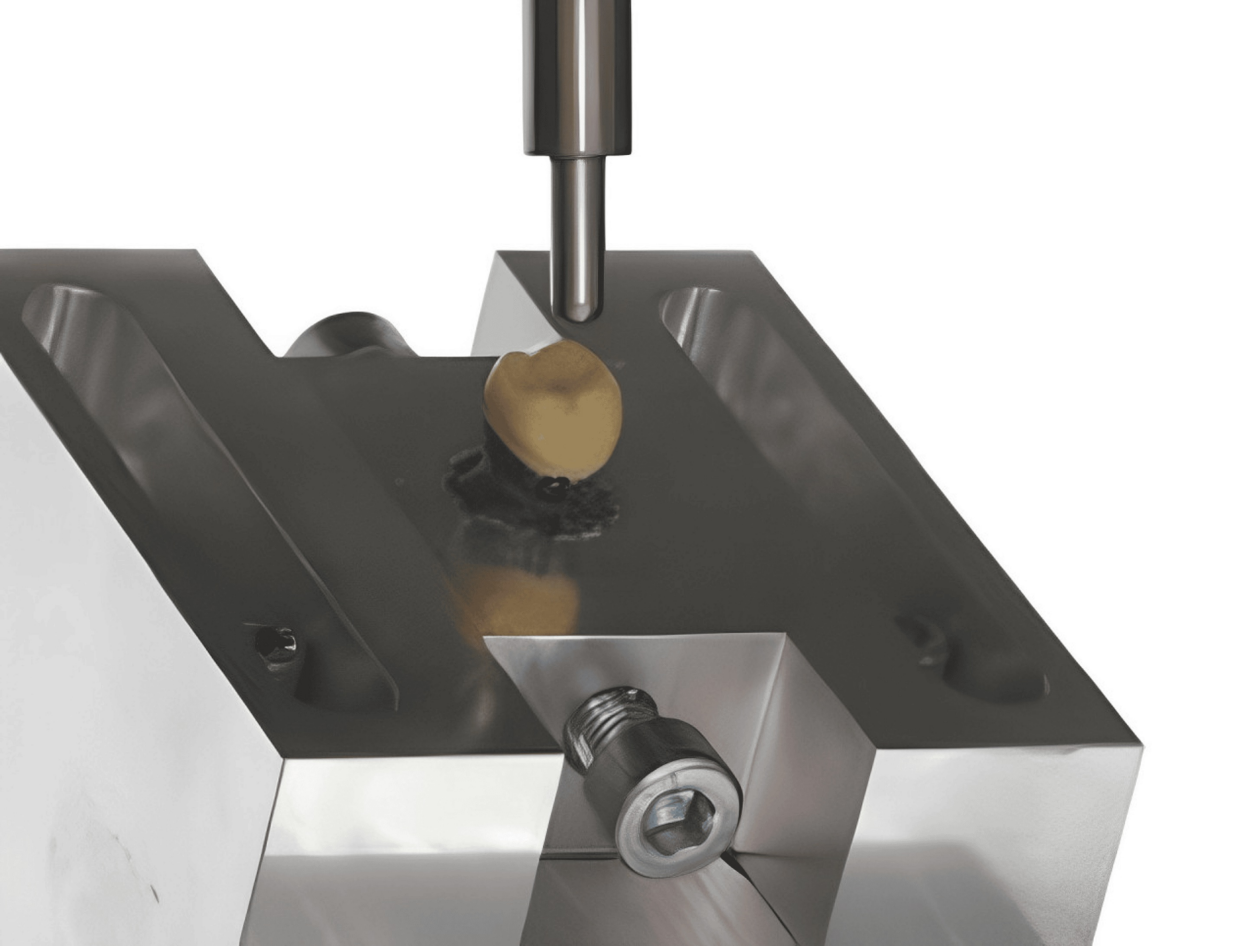
Universal testing machine to test the strength

In this study, a bonding strength test is done using a universal testing machine used to measure the adhesive characteristics of different dental cements on prepared teeth surfaces. Such machines offer precise control over testing conditions making it possible to accurately measure forces and displacements involved during testing. Each of the prepared tooth specimens has a respective dental cement layer under investigation (RMGIC, ZPC, or RC). These are then mounted securely on grips within a universal testing machine using specialized fixtures or clamps so that they cannot move around throughout the test. This ensures stability and consistency of specimen positioning within the machine while still allowing for variations between operators in different laboratories worldwide (Figure 3).

Once mounted with the required force applied through them by means of pulling along the longitudinal axis toward the direction opposite roots where they were extracted from earlier mentioned stumps, care must be taken not to apply excessive loads as this could lead to weak points in these samples. During such an arrangement, tensile stresses are created at the interface between dental cements and teeth surfaces. Finally, some form of failure like debonding or complete detachment occurs depending mainly upon the type of material used that bonds two different materials together.

The bond may break before reaching maximum pull force due to other reasons like surface contamination and poor adhesion quality, among others, but ideally, it should hold until a given moment when the desired level has been achieved, hence representing the highest strength recorded during such test under consideration thus far which is known as bonding strength measurement value; this can only be done if all necessary factors have been strictly adhered to throughout the execution period; otherwise, false data will be collected instead.

Marginal adaptation assessment

Evaluation of dental cement is incomplete until its ability to create a tight seal at the interface between a tooth and cement is tested, i.e., marginal adaptation evaluation. To carry out this evaluation, a stereo microscope was used, providing detailed views of structures in three dimensions and magnifying power-up magnification levels, ranging from 10× to 50×. Every tooth specimen prepared with a layer of dental cement for investigation was examined individually under a stereomicroscope. The focus was on the cement-tooth interface, where marginal adaptation is crucial in preventing microleakage and ensuring the long-term success of restoration. The examination involved checking the entire circumference of the cement-tooth interface for any gaps, discrepancies, or irregularities that could compromise the marginal seal. A stereomicroscope was used to magnify small details to detect even slight deviations from ideal marginal adaptation.

Marginal adaptation was assessed qualitatively by looking for gaps or discrepancies, which acted as indirect signs of how well cement worked. According to prosthodontic experts’ criteria, each sample got scores according to its level of marginal adaptation quality; higher numbers represented better performance characterized by close-fitting margins without breaks.

The scoring system used allowed for objective and uniform grading across all tested materials, regardless of their nature or origin. Scoring numerically made it possible to compare and quantify differentials between various dental cements regarding their ability to create the best possible seals against bacteria infiltration.

Assessment of microleakage

Microleakage appraisal plays an integral role in determining whether or not a given dental cement seals effectively at its interface with teeth. The methylene blue dye penetration method was employed here since it is widely recognized as one of the most reliable techniques for evaluating fluid ingress along the tooth-cement boundary.

For this purpose, each tooth specimen treated with a specific type(s) of adhesive material(s) was immersed into a penetrating solution while still mounted on a holder. Methylene blue dye, which has a high affinity toward small gaps, is capable of producing conspicuous color contrast following entry into them through capillarity action facilitated by gravity force attraction between molecules of liquid and solid surfaces having different wettabilities such as those presented at interfaces within restorative systems commonly used in dentistry practice today. Tooth specimens were immersed in methylene blue dye solution for 24 hours. After immersion, the samples were withdrawn and rinsed with distilled water for five minutes to remove excess surface stains. They were then air-dried for 10 minutes to eliminate residual moisture. Finally, the specimens were cut longitudinally with a precision saw to expose the cement-tooth junction, ensuring the integrity of the interface for accurate microscopic examination.

The cut sections were visualized under a microscope equipped with appropriate magnification power to observe the extent of penetration achieved by methylene blue dye at various points along the cement-tooth interface. Here, close attention was paid to detecting regions where this fluid had crept into spaces between different layers constituting dental filling, suggesting the presence or absence of a condition commonly referred to as secondary caries, which might signify marginal leakage indicative of failure of such materials establishing tight seals against microorganisms.

**Figure 4:**
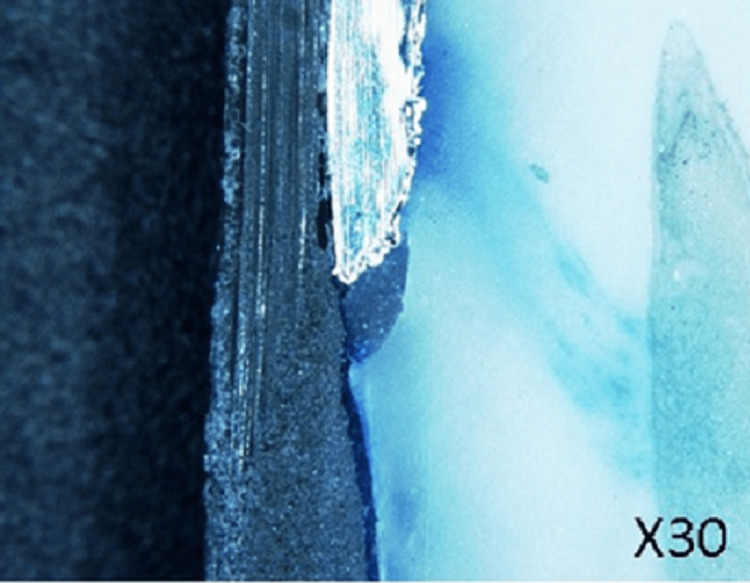
Microleakage testing by dye penetration under a microscope

The microscopic examination provides a comprehensive assessment of how well dental cement seals restorative interfaces, as it gives more information about where leaks occur than other methods do. Such areas showing entry should be quantitatively measured so that different types can be compared, while still being able to identify weak spots needing reinforcement if necessary (Figure 4).

The statistical package for social science (SPSS version 24, IBM Corp., Chicago, IL) was used to analyze data collected through checks on how strongly materials bonded and their adaptation at the edges as well as assessment of microleakages. For each dental cement variety, these outcomes were summarized using descriptive statistics, which included averages, range values, and standard deviation. Again, inferential statistics such as t-tests or analysis of variance (ANOVA) were applied in comparing results between various kinds of dental cement while p < 0.05 represented a statistically significant difference.

## Results

The bonding strength testing results indicated variations in the mean bonding strength among the different types of dental cement. RC exhibited the highest mean bonding strength (24.8 MPa, followed by RMGIC with a mean of 20.5 MPa and ZPC with a mean of 18.9 MPa). The ANOVA test revealed a statistically significant difference in bonding strength among the three types of dental cement (F(2, 45) = 4.72, p = 0.015), suggesting that the choice of cement significantly influences bonding strength outcomes (Table 1).

**Table 1:** Bonding strength testing results p < 0.05 is considered significant RMGIC: resin-modified glass ionomer cement; ZPC: zinc phosphate cement; RC: resin cement

Dental cement	Mean bonding strength (MPa)	Standard deviation (MPa)	Range (MPa)
RMGIC	20.5	2.3	18.2-23.8
ZPC	18.9	1.8	16.5-21.4
RC	24.8	3.1	21.5-28.3

The marginal adaptation evaluation results demonstrate differences in the mean scores representing the marginal adaptation quality across dental cements. RC exhibited the highest mean score of 4.0 mm, indicating superior marginal adaptation, followed by ZPC with a mean score of 3.2 mm and RMGIC with a mean score of 2.5 mm. The ANOVA test revealed a statistically significant difference in marginal adaptation scores among the three types of dental cement (F(2, 45) = 7.89, p = 0.003), indicating that the choice of cement significantly affected marginal adaptation (Table 2).

**Table 2:** Marginal adaptation evaluation results p < 0.05 is considered significant RMGIC: resin-modified glass ionomer cement; ZPC: zinc phosphate cement; RC: resin cement

Dental cement	Mean score (mm)	Standard deviation (mm)	Range (mm)
RMGIC	2.5	0.6	1-4
ZPC	3.2	0.8	2-5
RC	4.0	0.5	3-5

In the microleakage assessment results, variations in the mean dye penetration depth were observed among dental cements. ZPC exhibited the highest mean dye penetration depth (0.31 mm), followed by RMGIC with a mean of 0.25 mm and RC with a mean of 0.20 mm. The ANOVA test revealed a statistically significant difference in dye penetration depth among the three types of dental cement (F(2, 45) = 5.61, p = 0.008), suggesting that the choice of cement significantly influences microleakage (Table 3).

**Table 3:** Microleakage assessment results p < 0.05 is considered significant RMGIC: resin-modified glass ionomer cement; ZPC: zinc phosphate cement; RC: resin cement

Dental cement	Mean dye penetration depth (mm)	Standard deviation (mm)	Range (mm)
RMGIC	0.25	0.08	0.15-0.35
ZPC	0.31	0.06	0.25-0.40
RC	0.20	0.05	0.15-0.25

## Discussion

Structural support, sealing, and aesthetic improvement are among the numerous functions of dental cement in prosthodontic restorations. This study aimed to evaluate and compare the bond strength, marginal adaptation, and microleakage of three commonly used dental cements: RMGIC, ZPC, and RC.

A wide variety of materials used in prosthodontics necessitated selecting different types to encompass the entire field. Therefore, we had to decide what kind should be employed during this research work. Three categories, namely, RMGIC, ZPC, and RC, represented various groups based on compositionality and clinical applicability levels.

RMGICs combine characteristics seen in glass ionomers, such as fluoride release ability and chemical adhesion with those provided by resins, which improve physical properties plus esthetics; thus, they chemically bond to tooth structures, making them suitable for different types of crowns, bridges, orthodontic appliances. ZPC has been included since time immemorial because it serves as one traditional luting agent with some unique features apart from other cementations used in prosthodontics. ZPC shows great compressive strength together with low solubility; hence, it can be employed when fixing metal-based restorations like cast metal crowns and bridges; even though adhesive capabilities are limited compared with newer ones, it still remains an essential component due to its reliability as well as durability. RC classes were chosen because, among their kind, they are most preferred by dentists worldwide owing to superior bonding strengths coupled with high esthetics. Resin types of cement can bond strongly to both tooth structure and restorative materials. Hence, they are very useful for cementing all-ceramic, composite, or metal-free restorations. In addition, they cure fast and completely under light activation, thereby facilitating quick, long-term restoration procedures at the chairside [2,3].

No restoration can last long without the adequate bonding intensity of dental cement. In the present research, the mean bond strength was found to be highest in RC, followed by RMGIC and ZPC. This finding is consistent with other studies, which showed that resins have a higher ability to form strong bonds [13]. The high bonding capability of RC could be attributed to the micromechanical adhesion between it and both hard tooth substances as well as restorative materials, which is facilitated by the polymerization of resin monomers upon activation by light [14]. Composition and mode of adhesion may account for the relatively weaker adhesiveness exhibited by RMGIC or ZPC. While mechanical interlocking takes center stage during attachment between ZPCs and tooth structures, a combination of chemical stickiness and reinforcement through resins forms the basis for RMGICs' linkage [15]. It should, however, be noted that despite significant differences in bond strengths among these cements, this factor alone cannot be relied upon when selecting them for use in prosthodontics because there are some patient-specific concerns that need consideration, too.

Marginal adaptation is important since poor adaptations may lead to microleakages, which can cause secondary caries or even periodontal diseases around dental restorations. According to this study, the average score for marginal adaptation was highest with RC, followed by ZPC and RMGIC. These findings are similar to prior investigations, which reported that composites flow much better than other materials due to their low viscosity levels and thin film thicknesses [8,16]. Restoration margins get sealed tightly by RC, reducing the chances of having gaps that would compromise integrity at these sites while still ensuring a perfect fit. Marginal adaptation for RMGIC or ZPC ought to be satisfactory, but it may not always work depending on factors like the material’s flowability, shrinkage during polymerization, and sensitivity to different techniques [9]. Different clinical situations may, therefore, require clinicians to consider variations in these areas when choosing which cement to use during crown cementation or core build-ups.

Microleakage refers to the occurrence of secondary caries and pulpal inflammations due to fluids seeping along restorative margins containing microorganisms. The dye penetration technique was used in this study, where the mean depth of dye penetration recorded the highest values for ZPC, followed by RMGIC and RC, respectively. These results are consistent with what has been documented in the literature, showing that conventional cements like ZPC can experience increased levels of leakage mainly because they lack strong adhesiveness and are highly susceptible to moisture contamination [17]. On the other hand, resin-based cements offer better resistance against microleakages due to their chemical bonding properties and reduced water sorption [18]. Nevertheless, one needs caution while interpreting these findings since there are a number of variables affecting leakage, including the viscosity of the cement used and the surface preparation methods employed during the bonding process, among others, as well as aging effects [8].

Prosthetic practitioners should adopt the conclusions of this research as they are helpful in their work. It highlights the importance of customizing cement selection, examining the base for prostheses, patient-focused care, and following instructions already set. Restoration type, patients’ preferences, and characteristics of substrates are some of the things that should be considered when selecting dental cement for a given case. RCs are recommended when you need esthetics with strong bonding but less microleakage. At the same time, zinc phosphate may serve well on temporary or provisional restorations due to its cheapness and ease of use during the fixing process. Involving patients in shared decision-making and ensuring technique protocols are strictly followed can help minimize clinical failures associated with poor performance among different brands used in dentistry. Therefore, what clinicians need to do involves using these strategies based on available evidence coupled with being updated about emerging trends concerning cementation methods so that they deliver quality services centered around people’s needs, thus achieving the best outcome possible in terms of prosthodontic rehabilitation.

More study is necessary into new approaches to cementing materials, such as advanced formulations or bonding techniques, together with other supporting substances aimed at improving the service life span and overall functional efficiency of restorative procedures performed within the dental practice setting. Researchers may also wish to find out how various factors, like curing methods, affect bond strength between different types of cement used for filling teeth while considering marginal adaptation rates and leakage patterns over time among various groups treated by operators with varying levels of skills working in dissimilar environments and caring for diverse populations. There is a need for long-term follow-up research conducted over many years comparing success rates achieved after applying various brands of tooth restoration cement among different patients so that we can have confidence in our choice when deciding which product works better than the other, depending on individual circumstances. Collaboration between materials science specialists, dental researchers, and practitioners will greatly contribute toward the realization of good outcomes through improved care delivery systems within prosthodontics.

A key limitation of this study is the relatively short duration of observation, which may not fully capture the long-term performance and potential degradation of the dental cement. Additionally, variations in operator technique and environmental conditions during cement application could affect the consistency of results.

## Conclusions

In conclusion, the comprehensive assessment of dental cements conducted in this study offers valuable insights into their bonding strength, marginal adaptation, and microleakage properties, which are crucial factors that influence the success of prosthodontic restorations. Although RC exhibits superior bonding strength and marginal adaptation compared with other cement types, it is essential to recognize that each cement variant presents distinct advantages and limitations. Clinicians must carefully weigh these considerations along with patient-specific factors and clinical requirements when making informed decisions regarding cement selection for prosthodontic procedures. By incorporating evidence-based principles and prioritizing patient-centered care, clinicians can optimize treatment outcomes and enhance patient satisfaction with restorative treatments.

## References

[1] D'Souza D, Dua P (2011). Rehabilitation strategies for partially edentulous-prosthodontic principles and current trends. Med J Armed Forces India.

[2] Paul J (2015). Dental cements - a review to proper selection. Int J Curr Microbiol App Sci.

[3] Sirisha K, Rambabu T, Shankar YR, Ravikumar P (2014). Validity of bond strength tests: a critical review: part I. J Conserv Dent.

[4] Fan-Chiang YS, Chou PC, Hsiao YW (2023). Optimizing dental bond strength: insights from comprehensive literature review and future implications for clinical practice. Biomedicines.

[5] Abhishek G, Vishwanath SK, Nair A, Prakash N, Chakrabarty A, Malalur AK (2022). Comparative evaluation of bond strength of resin cements with and without 10-methacryloyloxydecyl dihydrogen phosphate (MDP) to zirconia and effect of thermocycling on bond strength - an in vitro study. J Clin Exp Dent.

[6] Ayyildiz S, Emir F, Pak Tunc E, Sen D (2015). Shear bond strength of various luting cements to fixed prosthodontic restorative materials. Appl Adhes Sci.

[7] Badami V, Satya Priya M, Vijay L, Kethineni H, Akarapu S, Agarwal S (2022). Marginal adaptation of veneers: a systematic review. Cureus.

[8] Bagheri R (2013). Film thickness and flow properties of resin-based cements at different temperatures. J Dent (Shiraz).

[9] Parameswari BD, Rajakumar M, Lambodaran G, Sundar S (2016). Comparative study on the tensile bond strength and marginal fit of complete veneer cast metal crowns using various luting agents: an in vitro study. J Pharm Bioallied Sci.

[10] Singh S (2023). Microleakage studies - a viewpoint. J Conserv Dent.

[11] Da Silva D, Ceballos L, Fuentes MV (2021). Influence of the adhesive strategy in the sealing ability of resin composite inlays after deep margin elevation. J Clin Exp Dent.

[12] Ladha K, Verma M (2010). Conventional and contemporary luting cements: an overview. J Indian Prosthodont Soc.

[13] Heboyan A, Vardanyan A, Karobari MI (2023). Dental luting cements: an updated comprehensive review. Molecules.

[14] Maletin A, Knežević MJ, Koprivica DĐ, Veljović T, Puškar T, Milekić B, Ristić I (2023). Dental resin-based luting materials-review. Polymers (Basel).

[15] Mitchell CA, Abbariki M, Orr JF (2000). The influence of luting cement on the probabilities of survival and modes of failure of cast full-coverage crowns. Dent Mater.

[16] Baltacioğlu İH, Demirel G, Öztürk B, Aydin F, Orhan K (2024). Marginal adaptation of bulk-fill resin composites with different viscosities in class II restorations: a micro-CT evaluation. BMC Oral Health.

[17] Jacob J, Devadathan A, Joseph S, Dathan PC, Mathew S, Kuriakose R (2022). Comparative evaluation of microleakage of zinc phosphate cement, resin-modified glass ionomer, and two dual-cure resin cements: in vitro study. J Pharm Bioallied Sci.

[18] El-Mowafy O (2001). The use of resin cements in restorative dentistry to overcome retention problems. J Can Dent Assoc.

